# Characterization of shade tolerance gene network in soybean revealed by forward integrated reverse genetic studies

**DOI:** 10.1093/hr/uhae333

**Published:** 2024-11-26

**Authors:** Yanzhu Su, Yongpeng Pan, Weiying Zeng, Zhenguang Lai, Pengfei Guo, Xiaoshuai Hao, Shengyu Gu, Zhipeng Zhang, Lei Sun, Ning Li, Jianbo He, Wubin Wang, Guangnan Xing, Jiaoping Zhang, Zudong Sun, Junyi Gai

**Affiliations:** Soybean Research Institute & MARA National Center for Soybean Improvement & MARA Key Laboratory of Biology and Genetic Improvement of Soybean & State Key Laboratory for Crop Genetics and Germplasm Enhancement & State Innovation Platform for Integrated Production and Education in Soybean Bio-breeding & Jiangsu Collaborative Innovation Center for Modern Crop Production, Nanjing Agricultural University, Nanjing, Jiangsu 210095, China; Soybean Research Institute & MARA National Center for Soybean Improvement & MARA Key Laboratory of Biology and Genetic Improvement of Soybean & State Key Laboratory for Crop Genetics and Germplasm Enhancement & State Innovation Platform for Integrated Production and Education in Soybean Bio-breeding & Jiangsu Collaborative Innovation Center for Modern Crop Production, Nanjing Agricultural University, Nanjing, Jiangsu 210095, China; Institute of Economic Crops, Guangxi Academy of Agricultural Sciences, Nanning, Guangxi 530007, China; Institute of Economic Crops, Guangxi Academy of Agricultural Sciences, Nanning, Guangxi 530007, China; Soybean Research Institute & MARA National Center for Soybean Improvement & MARA Key Laboratory of Biology and Genetic Improvement of Soybean & State Key Laboratory for Crop Genetics and Germplasm Enhancement & State Innovation Platform for Integrated Production and Education in Soybean Bio-breeding & Jiangsu Collaborative Innovation Center for Modern Crop Production, Nanjing Agricultural University, Nanjing, Jiangsu 210095, China; Soybean Research Institute & MARA National Center for Soybean Improvement & MARA Key Laboratory of Biology and Genetic Improvement of Soybean & State Key Laboratory for Crop Genetics and Germplasm Enhancement & State Innovation Platform for Integrated Production and Education in Soybean Bio-breeding & Jiangsu Collaborative Innovation Center for Modern Crop Production, Nanjing Agricultural University, Nanjing, Jiangsu 210095, China; Soybean Research Institute & MARA National Center for Soybean Improvement & MARA Key Laboratory of Biology and Genetic Improvement of Soybean & State Key Laboratory for Crop Genetics and Germplasm Enhancement & State Innovation Platform for Integrated Production and Education in Soybean Bio-breeding & Jiangsu Collaborative Innovation Center for Modern Crop Production, Nanjing Agricultural University, Nanjing, Jiangsu 210095, China; Soybean Research Institute & MARA National Center for Soybean Improvement & MARA Key Laboratory of Biology and Genetic Improvement of Soybean & State Key Laboratory for Crop Genetics and Germplasm Enhancement & State Innovation Platform for Integrated Production and Education in Soybean Bio-breeding & Jiangsu Collaborative Innovation Center for Modern Crop Production, Nanjing Agricultural University, Nanjing, Jiangsu 210095, China; Soybean Research Institute & MARA National Center for Soybean Improvement & MARA Key Laboratory of Biology and Genetic Improvement of Soybean & State Key Laboratory for Crop Genetics and Germplasm Enhancement & State Innovation Platform for Integrated Production and Education in Soybean Bio-breeding & Jiangsu Collaborative Innovation Center for Modern Crop Production, Nanjing Agricultural University, Nanjing, Jiangsu 210095, China; Soybean Research Institute & MARA National Center for Soybean Improvement & MARA Key Laboratory of Biology and Genetic Improvement of Soybean & State Key Laboratory for Crop Genetics and Germplasm Enhancement & State Innovation Platform for Integrated Production and Education in Soybean Bio-breeding & Jiangsu Collaborative Innovation Center for Modern Crop Production, Nanjing Agricultural University, Nanjing, Jiangsu 210095, China; Soybean Research Institute & MARA National Center for Soybean Improvement & MARA Key Laboratory of Biology and Genetic Improvement of Soybean & State Key Laboratory for Crop Genetics and Germplasm Enhancement & State Innovation Platform for Integrated Production and Education in Soybean Bio-breeding & Jiangsu Collaborative Innovation Center for Modern Crop Production, Nanjing Agricultural University, Nanjing, Jiangsu 210095, China; Soybean Research Institute & MARA National Center for Soybean Improvement & MARA Key Laboratory of Biology and Genetic Improvement of Soybean & State Key Laboratory for Crop Genetics and Germplasm Enhancement & State Innovation Platform for Integrated Production and Education in Soybean Bio-breeding & Jiangsu Collaborative Innovation Center for Modern Crop Production, Nanjing Agricultural University, Nanjing, Jiangsu 210095, China; Soybean Research Institute & MARA National Center for Soybean Improvement & MARA Key Laboratory of Biology and Genetic Improvement of Soybean & State Key Laboratory for Crop Genetics and Germplasm Enhancement & State Innovation Platform for Integrated Production and Education in Soybean Bio-breeding & Jiangsu Collaborative Innovation Center for Modern Crop Production, Nanjing Agricultural University, Nanjing, Jiangsu 210095, China; Soybean Research Institute & MARA National Center for Soybean Improvement & MARA Key Laboratory of Biology and Genetic Improvement of Soybean & State Key Laboratory for Crop Genetics and Germplasm Enhancement & State Innovation Platform for Integrated Production and Education in Soybean Bio-breeding & Jiangsu Collaborative Innovation Center for Modern Crop Production, Nanjing Agricultural University, Nanjing, Jiangsu 210095, China; Institute of Economic Crops, Guangxi Academy of Agricultural Sciences, Nanning, Guangxi 530007, China; Soybean Research Institute & MARA National Center for Soybean Improvement & MARA Key Laboratory of Biology and Genetic Improvement of Soybean & State Key Laboratory for Crop Genetics and Germplasm Enhancement & State Innovation Platform for Integrated Production and Education in Soybean Bio-breeding & Jiangsu Collaborative Innovation Center for Modern Crop Production, Nanjing Agricultural University, Nanjing, Jiangsu 210095, China

## Abstract

Shade tolerance is a key trait for cultivars in inter/relay-cropped soybeans in maize fields. Our previous genome-wide association study (GWAS) results on southern China soybean germplasm revealed that the shade tolerance was conferred by a complex of genes with multiple alleles. To complete our understanding of the shade tolerance gene system, GWAS with gene–allele sequences as markers (designated GASM-RTM-GWAS) was conducted in a recombinant inbred line (RIL) population between two extreme parents using the shade tolerance index (STI) and relative pith cell length (RCL) as indicators. Altogether, 211 genes, comprising 99 and 119 genes (seven shared) for STI and RCL, respectively, were identified and then annotated into a similar set of five biological categories. Furthermore, transcriptome analysis detected 7837 differentially expressed genes (DEGs), indicating plentiful DEGs involved in the expression of regulatory/causal GWAS genes. Protein–protein interaction (PPI) analysis and gene functional analysis for both GWAS genes and DEGs showed a group of interrelated causal genes and a group of interrelated DEGs; the former were included in the latter and their functions were interconnected as a gene network. For further understanding of the response of soybean to shade stress in a sequential connection, six chronological gene modules were grouped as signal activation and transport, signal-transduction, signal amplification, gene expression, regulated metabolites, and material transport. From the modules, 12 key genes were selected as entry points for further analysis. Our study provides an overview of the shade tolerance gene network as a new insight into a complex-trait genetic system, rather than the usual way of starting from a hand-picked single gene.

## Introduction

Chinese farmers have developed an innovative cropping system of inter/relay-cropped soybean (including vegetable soybean) in maize fields for an additional soybean harvest from a single piece of land [1–4]. In this cropping system, the soybean plants may suffer from shading stress during their symbiosis with maize, resulting in decreased biomass, stem diameter, leaf area and thickness, and stem strength [5, 6], and increased plant height, lodging score and stem twining [7, 8], finally causing yield limitation [9]. To evaluate the shade tolerance of soybeans, Sun *et al*. [10] used the shade-tolerance index (STI) under shading versus non-shading stress as a morphological indicator, while Su *et al*. [11] used relative pith cell length (RCL) under shading versus non-shading stress as an internal anatomical indicator. The smaller the STI and RCL, the stronger the shade tolerance.

To explore the shade tolerance genetic system in a soybean–maize inter/relay-cropping system under simulated conditions, a genetic study [12] on lignin contents was conducted in recombinant inbred lines (RILs) using inclusive composite interval mapping (ICIM) in the IciMapping 4.1 procedure, in which 13 quantitative trait loci (QTLs) were detected, but only one gene was predicted (*Glyma.01 g169200*). Recently, 63 STI genes with their 308 alleles were identified in a southern China soybean germplasm population using a restricted two-stage multilocus model genome-wide association study (RTM-GWAS) with gene–allele sequences as markers (GASM, GASM-RTM-GWAS) under 30% reduction of sunlight in a net room [13]. The study concluded that a group of genes with different biological functions conferred shade tolerance; therefore, this is a complex trait rather than an oligogenic one. Furthermore, using GASM-RTM-GWAS with STI and RCL as indicators in integrated transcriptomic analysis for an RIL population [11], the GWAS identified 140 and 146 genes with 9 of genes shared for the two indicators, respectively, while the transcriptome identified 94 differentially expressed genes (DEGs) shared by the GWAS. These previous studies confirmed the genetically complex concept of the trait.

In complex traits, a large number of genes work in a series of coordinated functions, but in fact the basic genetic unit is an allele of a gene; these are usually referred to together as a gene system. This is quite different from the oligogenic situation, in which it is necessary only to screen for best accession(s) with the target gene/allele(s) and then to identify and clone the gene/allele through Mendelian procedures. In previous research [11], both STI and RCL gene systems involving a similar set of biological functions were interrelated through protein–protein interaction (PPI) analysis. These different gene systems compose a gene network corresponding to the shade tolerance of soybean. But it is yet to be clarified whether a gene network is only a concept or a defined entity of genes and whether or how genes with different functions are interrelated.

To discuss the gene network of a trait, it is a prerequisite that the genes and their alleles can be identified thoroughly. As indicated above, the innovative GASM-RTM-GWAS is qualified to do so for a germplasm population using GASM to replace the genomic marker SNPLDB (SNP linkage disequilibrium block) (https://github.com/njau-sri/rtm-gwas or https://gitee.com/njau-sri/rtm-gwas) [11, 13, 14]. The use of GASM benefits by directly identifying the genes with their alleles without requirement to infer candidate genes from SNPLDBs. In comparison with the widely used maximum likelihood GWAS methods, the RTM-GWAS is characterized by two innovations. The first is the use of multiple allele markers to fit the requirement of multiple alleles in the germplasm population or breeding population; the second is to minimize false-positives and false-negatives through a series of techniques. These include a multilocus model to control the influence of neighboring loci with a reasonable model test significance level and total contribution less than *h*^2^, two-stage analysis to reduce noise from excessive markers, the top 10 eigenvectors as covariates for population structure correction, and precise experimental design. The advantages of this method have been validated in a series of studies [11, 13–19].

Based on GWAS, the identified genes are annotated into biological categories to understand their functional relationships using the accumulated knowledge in SoyBase (http://www.soybase.org). This classification can be further checked using the Phytozome database (http://phytozome.jgi.doe.gov) to transfer the gene information into the protein database, then using the STRING procedure (http://string-db.org) to generate the PPI network. These are forward genetic analyses. On the other hand, the genetic system can be explored through reverse genetic procedures, i.e. transcriptome analysis to identify the DEGs. Further analysis can provide insight into the changes in DEG expression level, important biological processes, and pathways. Forward integrated with reverse genetic analysis has been applied successfully to analyze the complete genetic system of traits and to identify key candidate genes in various crops [20–25]. However, of the forward and reverse genetic analyses, the former should be the base for genetic system analysis because the causal genes from GWAS will activate their expression processes.

In the present study, our aim was to further explore and characterize the gene network of shade tolerance for soybean as an entity, with their interrelationship revealed. In doing so, our plan was as follows. (i) To simplify the genetic background, an RIL population from a pair of extreme parents is to be used. (ii) The first step is to identify all the regulatory/causal STI and RCL genes with their alleles through a forward genetic procedure using GASM-RTM-GWAS. (iii) The functions of the GWAS-identified genes are annotated and their interaction is to be explored using GWAS-PPI analysis. (iv) The expressed gene system is explored through transcriptome analysis. (v) The functions of the DEGs are to be annotated through the Mercator4 procedure and their interaction are to be explored through DEG-PPI. (vi) The similarity and dissimilarity between the forward and reverse genetic results will be compared to summarize the STL and RCL versus transcriptome gene system. (vii) To predict a shade tolerance gene module series involved in chronological order from the initial signal perception to the final response to shade stress. Then, the key shade tolerance genes may be selected from the modules for further detailed studies.

## Results

### The external and internal indicators shade tolerance index and relative pith cell length provide a unified shade tolerance evaluation for the GZ-RIL population

**Figure 1:**
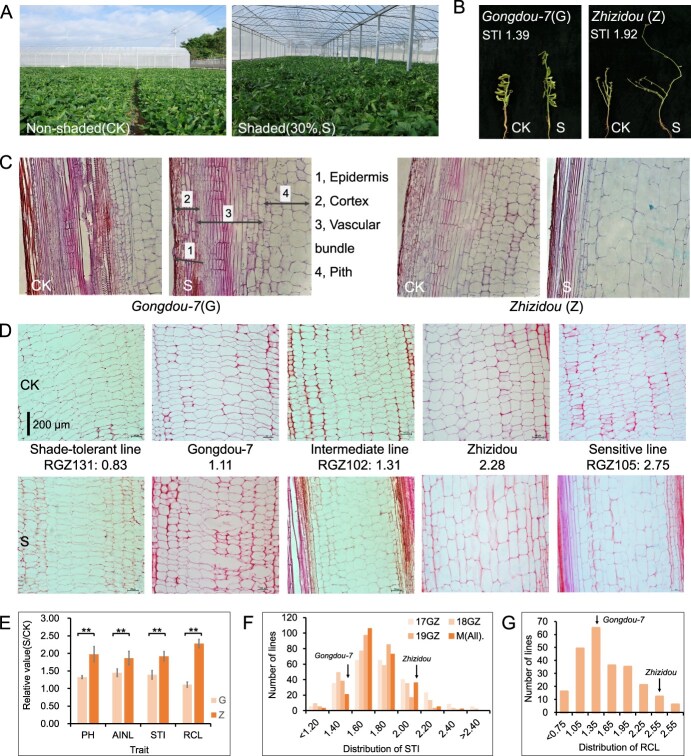
Morphological differences in the two parents and GZ-RIL population under control (CK) and shade stress. **A** Testing design of the shaded and non-shaded experiment. **B** Phenotypes of the two parents evaluated on day 50 after sowing. **C** Cell images of the main stem penultimate internode for the two parents under light microscopy (10×) on day 50 after sowing. **D** Cell images of the pith tissue (main stem penultimate internode) for two parents and lines under light microscopy (10×) on day 50 after sowing. RGZ131, RGZ102, and RGZ105 are the lines in the GZ-RIL population and 0.83, 1.11, etc. are the average RCL values. Graph colors are not uniform due to different experimental batches and microscope observation times. **E** Relative phenotypic values of various traits of the parents on day 50 after sowing under two conditions. PH and AINL are plant height and average internode length, respectively. ^**^P < 0.01. **F** Frequency distribution of STI in the GZ-RIL population under three environments, in which 17GZ, 18GZ, and 19GZ indicate that the GZ-RIL population was planted in 2017, 2018, and 2019, respectively, while M(all) means the average over the three environments. **G** Frequency distribution of RCL in the GZ-RIL population under one environment.

Under shade stress, Gongdou-7 had a significantly lower plant height and internode length plus stronger and harder stems compared with Zhizidou, especially the upper internode (Fig. 1A and B). Optical microscopy observations revealed that the elongation rate of penultimate internode cells in Gongdou-7 under shading was obviously lower than that of Zhizidou cells, especially that of pith cell length (Fig. 1C and 1D). Plant height (PH) and average internode length (AINL), STI and RCL values showed a very significant difference between the two parents (Fig. 1E, Supplementary Data Table S1).

Analysis of variance for the two indicators showed very significant variation among the RILs. The frequency distribution of STI and RCL in the GZ-RIL population ranged from 1.13 to 2.28 and from 0.44 to 3.05, respectively, indicating broad variation among RILs, especially for RCL (Fig. 1D, F and G and Supplementary Data Table S1). Large transgressive segregation existed in the RIL population for the two traits. A highly significant positive correlation (*r* = 0.54, *P* < 0.01) was observed between STI and RCL, which indicates that the two traits are a pair of synchronous indicators and may work jointly for shade tolerance in soybean. There existed significant RILs × environment interaction in STI, but this was not evaluated for RCL due to the large workload requirement for its measurement. The total heritability value (*h*^2^) of STI was 67.17% (main effect) + 18.02% (RILs × environment) = 85.19%, while that of RCL was 98.88% (with that of RILs × environment not separated); both heritability values were high enough for further identification of candidate genes in GASM-RTM-GWAS.

### Forward genetic study identified numerous shade tolerance index and relative pith cell length genes with their alleles as a shade tolerance gene–allele system through GASM-RTM-GWAS

**Figure 2:**
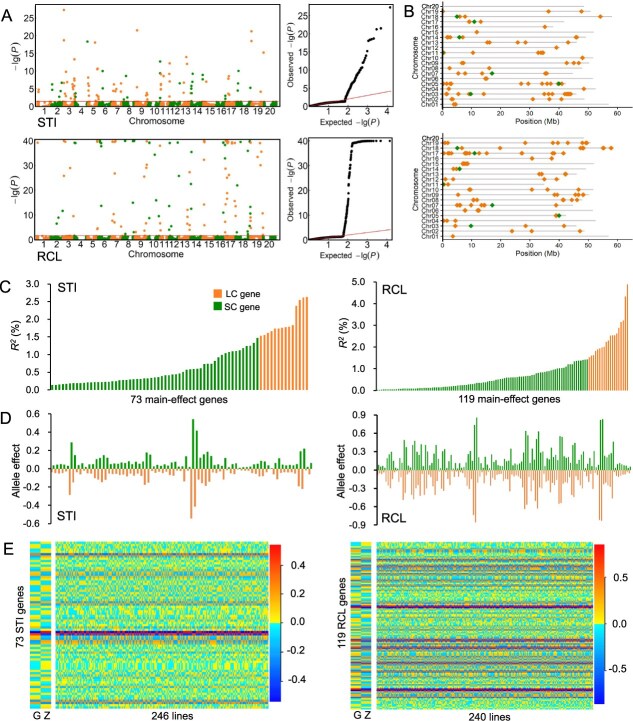
Identification of gene–allele system conferring shade tolerance in the GZ-RIL population. **A** Manhattan and Q–Q plots for STI and RCL using GASM-RTM-GWAS. −log *P* > 40 values are all shown as −log *P* = 40 in RCL. The red line on the Manhattan plot is the significance threshold, and the loci above the threshold are candidate genes detected by association analysis, including 99 STI and 119 RCL genes, respectively. **B** Distribution of associated genes for STI and RCL on soybean chromosomes. No significant gene was detected on chromosome 20. The seven green dots are shared genes between STI and RCL. **C** The phenotypic contributions of the 73 STI and 119 RCL main-effect genes that were detected. The vertical axis represents the genetic contribution to phenotypic variance (*R*^2^, %); the horizontal axis represents the main-effect genes, arranged in increasing order. **D** Allele effects of STI and RCL genes. The green and orange bars represent positive and negative alleles, respectively. **E** STI and RIL gene–allele matrices of the parents and RIL populations. The horizontal axis shows 246/240 RILs arranged in increasing order from left to right for STI and RCL values, respectively. The vertical axis is the allele effect expressed in color darkness, arranged in increasing chromosome order from bottom to top, where yellow indicates a positive value, blue indicates a negative value, and color darkness indicates a larger absolute value.

In the GZ-RIL population, 7103 GASMs were assembled from 83 407 SNPs, covering 20 chromosomes, each with 134–553 GASMs (Supplementary Data Fig. S1). At the first stage of GASM-RTM-GWAS, 2289 and 2790 GASMs were preselected for STI and RCL, respectively. At the second stage, 99 and 119 genes and their alleles (with allele effects) were identified for STI and RCL, respectively, including 7 genes shared by both indicators (Fig. 2A–D; Supplementary Data Tables S2 and S3).

For STI, 73 main-effect genes were identified and contributed 57.44% of the phenotypic variance (PV), each in the range 0.14–2.63% of PV, whereas 63 gene × environment interaction (GEI) genes explained 32.32% of PV, ranging from 0.22 to 1.37% of PV (Supplementary Data Table S4). For RCL, 119 main-effect genes contributed 96.42% of PV, ranging from 0.02 to 4.86%, but without GEI genes identified due to only a single environment being involved. Among the identified main-effect genes, 14 STI and 19 RCL genes made a large contribution (LC), explaining 27.15 and 47.66% of PV, respectively, while 59 STI and 100 RCL genes made a small contribution (SC), explaining 30.29 and 48.76% of PV (Supplementary Data Table S4). Here the boundary between LC and SC was around the inflection point of the QTL PV contribution curve. From the above, STI and RCL were both quantitatively inherited traits with continuously varying genetic contributions (Fig. 2C), in a total of 99 + 119–7 = 211 genes (7 genes shared by both traits). In addition, the total genetic contribution (*h*^2^) minus those of LC and SC genes is the genetic contribution due to a collection of unmapped minor-effect polygenes, which contribute 9.73 and 2.46% of PV for STI and RCL, respectively.

**Figure 3:**
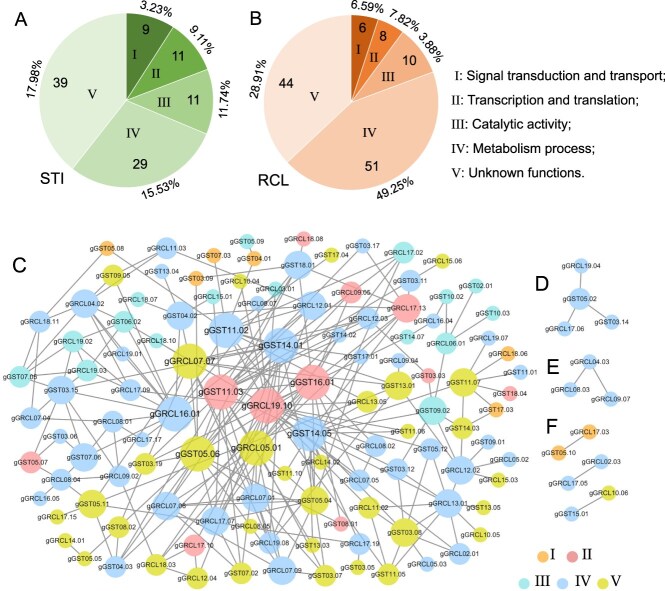
Functional categories and PPI network for STI and RCL GWAS genes. **A**, **B** Functional categories/groups (I–V) of STI and RCL genes. The Arabic numbers in the pie charts are the numbers of genes corresponding to each category/group. The numbers outside the pie chart represent the contribution of each group of main-effect genes. **C**–**F** Each node represents a gene/protein. The connecting lines (edges) between genes indicate that they interact, which means the two proteins jointly contribute to a shared function; this does not necessarily mean they physically bind to each other. Nodes from small to large represent different number of edges: 1–2, 3–5, 6–10, and 10–20. Five colored nodes correspond to functional categories/groups I–V.

All genes contain only two alleles in the GZ-RIL population because of only two parents. Here, the allele effects ranged from −0.54 to 0.54 for STI and − 0.86 to 0.86 for RCL, while the majority of the allele effects varied between −0.2 and 0.2 (Fig. 2D). The genes with their allele effects of the two indicators were organized into a gene–allele matrix (the allele effect expressed in color darkness in Fig. 2E), respectively, which was in fact composed of the entire genetic constitution of a trait in the GZ-RIL population. From the matrices, the recombination potential between two RILs in the population for STI and RCL were predicted. Total recombination potentials of 30 135/28 680 RIL pairs (crosses) among the 246/240 RILs were predicted for the STI/RCL trait, with the highest one as the best recombination potential for the biparental population. Here, using the 10th percentile as an indicator, the predicted values under the linkage model were 0.62 and − 0.06, and those under the independent assortment model were 0.33 and −2.38 for STI and RCL, respectively (Supplementary Data Table S4). A quite large transgressive advancement might be expected under the linkage model, while more progress can be expected under the independent assortment model. This means that under the linkage model some linkage obstacle may prevent further recombination (Supplementary Data Table S4).

### The identified shade tolerance index and relative pith cell length gene–allele systems involve a similar set of five biological function categories as a regulatory/causal gene system

In the annotation of the identified 99 STI and 119 RCL genes, they were grouped into the same set of five biological function categories using Gene Ontology (GO) analysis (Fig. 3A and B, Table 1, Supplementary Data Tables S2 and S3). These include the following categories: (I) signal transduction and transport (9 STI + 6 RCL), (II) transcription and translation in gene expression (11 + 8), (III) catalytic activity (11 + 10), (IV) primary metabolism and other biological process (29 + 51), and (V) unknown functions (39 + 44). GO annotation suggests that the survival of plants under shade stress depends on the recognition of environmental stimuli, signal generation and transmission, gene expression, and metabolic regulation; moreover, many unknown function genes in category V are also involved in important biological processes. STI and RCL gene systems and their distribution among the categories were very similar, both with a large part located in categories IV and V but with only seven genes shared. This indicates that the two gene systems share a similar constitution pattern, but for the most part the gene constitution was still different (Table 1).

**Table 1:** Statistics of GWAS-identified genes in five functional categories.

Item^a^	Gene^b^	I^c^		II		III		IV		V		Total	
		No.	*R* ^2^ (%)	No.	*R* ^2^ (%)	No.	*R* ^2^ (%)	No.	*R* ^2^ (%)	No.	*R* ^2^ (%)	No.	*R* ^2^ (%)
GWAS	STI (LC)	9	3.23	11 (4)	9.11 (7.54)	11 (4)	11.74 (9.53)	29 (3)	15.35 (4.98)	39 (3)	17.98 (5.10)	99 (14)	57.42 (27.13)
	RCL (LC)	6 (1)	6.59 (2.54)	8 (2)	7.82 (4.16)	10 (1)	3.88 (1.89)	51 (11)	49.25 (31.13)	44 (3)	28.91 (6.34)	119 (19)	96.46 (47.66)
	Shared	1		1		1		3		1		7	
PPI	STI (LC)	6	1.85	6 (2)	4.84 (4.17)	8 (3)	8.09 (6.90)	21 (2)	11.08 (3.21)	20 (1)	8.69 (1.74)	61 (8)	34.55 (16.20)
	RCL (LC)	3 (1)	3.06 (2.54)	5 (1)	4.27 (1.62)	8 (1)	3.60 (1.89)	41 (8)	40.83 (24.56)	15 (3)	12.29 (5.95)	72 (14)	64.05 (34.56)
	Shared	1						2		1		4	
	Key hub			3				5		3		11	
DEG	STI (LC)	1	0.19	2 (1)	2.38 (2.38)	2	0.73	5	0.58	5 (1)	3.17 (1.53)	15 (2)	7.15 (3.91)
	RCL (LC)			1	1.02	3	0.67	7 (1)	5.50 (3.23)	3	1.68	14 (1)	8.87 (3.23)
	Shared							2				2	
	PPI			3		5		8				16	

a In Item column, GWAS is the functional category distribution of STI and RCL genes in the GZ-RIL population; PPI is the functional category distribution of STI and RCL genes in the protein–protein interaction network.

b In the gene column LC represents a large contribution (*R*^2^ ≥ 1.5%) gene; Shared indicates the number of genes shared by both STI and RCL; Key hub indicates the number of key hub genes in the PPI network; a node gene is called a key hub gene when it interacts with more than 10 other genes (links ˃10).

c I–V are gene functional categories: I, signal transduction and transport genes; II, transcription and translation genes; III, catalytic activity genes; IV, primary metabolism and other biological process genes; V, Unknown function genes. No., number of genes.

To explore the interrelationship between the two sets of genes, the PPI was predicted (Fig. 3C−F) to be significant at *P* = 9.56E−10^*^, indicating that STI and RCL genes worked tightly as a regulatory/causal gene system for shade tolerance. Here, 129 (61%) genes were predicted in six PPI groups, including one with 4 genes (Fig. 3D), one with 3 genes (Fig. 3E), three with 2 genes (Fig. 3F), and one with the remaining 116 genes (55.0%), as the largest GWAS gene PPI group (Fig. 3C). This largest one included 61 STI and 72 RCL genes, with 22 (8 STI + 14 RCL) LC genes and 4 shared genes (Table 1, Supplementary Data Tables S2 and S3). These genes in the PPI groups were involved in all five functional categories (I–V), including categories I (6STI + 3RCL), II (6 + 5), III (8 + 8), IV (21 + 41), and V (20 + 15) (Table 1, Supplementary Data Tables S2 and S3). Among them, the number of genes in category IV was the maximum, indicating that, under shade stress, soybean plants had a large number of genes involved in metabolic processes under the synergistic promotion of signal transduction and transport, gene expression, and enzymes to maintain normal life activities. In addition, 6 STI and 5 RCL genes (including 1 shared gene) interacted frequently with other genes (links = 11–20), forming a close association as key hub genes. *gGST11.03* (STI) and *gGRCL19.10* (RCL) were the largest key hub genes with 20 links (Fig. 3C), encoding a structural constituent of ribosome (GO:0003735) and RNA polymerase II transcription cofactor activity (GO:0001104), related to transcription and translation (Supplementary Data Tables S2 and S3). This indicates that metabolic process is most active and complicated in the response to shade stress, while gene expression plays a core role.

From the above, the 217 STI and RCL genes, which are involved in a similar set of five functional categories and linked together, compose a regulatory/causal gene system in forward genetics.

### Reverse genetics for shade tolerance through transcriptome analysis identified extremely numerous differentially expressed genes

To further explore the DEGs under shade versus non-shade conditions between the two parents, Gongdou-7 and Zhizidou were subjected to transcriptomic analysis. After stringent quality filtering, the data showed superiority in quality and repeatability, with high correlation among replicates (Supplementary Data Fig. S2 and B; Supplementary Data Table S5). At the same time, the qRT–PCR results were highly consistent with those of RNA-sequencing data in the parents (Supplementary Data Fig. S2C), suggesting that these sample library data could be used for detailed genetic analysis.

**Figure 4:**
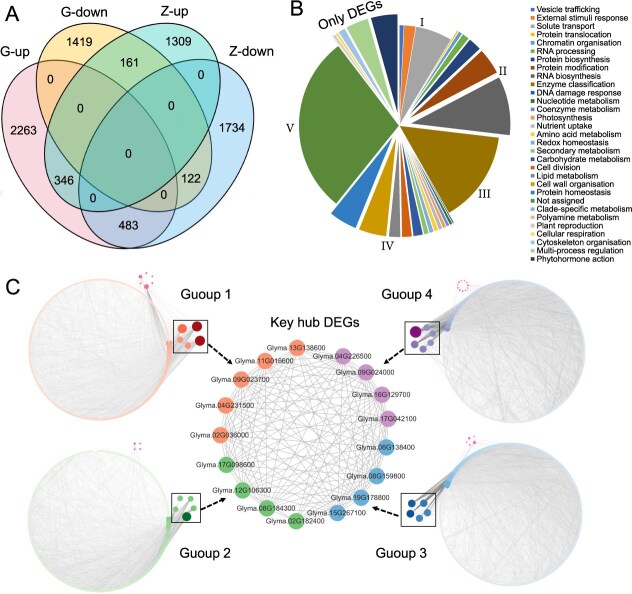
DEGs, their functional enrichment, and their PPI network construction between the two parents. **A** Venn diagram of DEGs. Number of DEGs identified in pairwise comparisons between shading (S) and non-shading (CK) conditions for the two parents on day 30. G-up, G-down, Z-up, and Z-down indicate upregulation in G_S30_ versus G_CK30_, downregulation in G_S30_ versus G_CK30_, upregulation in Z_S30_ versus Z_CK30_ and downregulation in Z_S30_ versus Z_CK30_, respectively. **B** Functional annotation of DEGs using Mercator4 and their correspondence with GWAS gene annotation. I–V are functional categories/groups for STI and RCL GWAS genes, indicating DEGs and GWAS-identified genes with a similar functional category. **C** PPI networks of DEGs (DEG-PPI) in two parents. Each node represents a DEG (protein). The 7837 DEGs were divided into four groups (Group 1, Group 2, Group 3, and Group 4), forming four PPI networks. The DEGs form a circle for each group. The nodes in squares are key hub DEGs; warm color nodes are DEGs from 26 GWAS genes. The large circle in the middle is a PPI network connecting key hub DEGs. The gray background lines in each circle are interaction lines between DEGs.

The RNA-sequencing analysis of the two parents on day 30 under shaded versus non-shaded conditions showed a total of 32 695 expressed genes identified (Supplementary Data Fig. S2D). Among them, 4794 DEGs (3092 upregulated + 1702 downregulated) were identified in G_S30_ versus G_CK30_ (Gongdou-7), and 4155 (1816 + 2339) DEGs in Z_S30_ versus Z_CK30_ (Zhizidou) (Fig. 4A); more DEGs were detected in Gongdou-7, suggesting that the shade-tolerant parent had more complicated regulatory mechanisms in shading acclimation than the shade-sensitive parent. Among the DEGs, 2263 upregulated and 1419 downregulated, a total of 3682 DEGs, existed only in G_S30_ versus G_CK30_, while 1309 upregulated and 1734 downregulated, a total of 3043 DEGs, were unique in Z_S30_ versus Z_CK30_. There is a difference of 873 (2263–1390) in the upregulated DEGs between the two parents, and of 315 (1734–1419) in the downregulated DEGs, so shade tolerance might be more likely related to upregulation and shade sensitivity to downregulation. In addition, 1112 DEGs were shared by the two parents, in which 644 (483 + 161) DEGs had opposite expression trends (qualitative differences), while 468 (346 + 122) DEGs had the same expression trend (quantitative differences) (Fig. 4A). This means a total of 3682 + 3043 + 1112 = 7837 DEGs were different between the two parents, leading to obvious differentiation in shade tolerance between the two parents.

### Reverse genetics showed that the shade tolerance-expressed gene system was composed of wider functional categories of genes compared with the GWAS-identified gene system

Mercator4 is an online tool that assigns plant protein sequences to functional categories based on sequence similarity relationships. To identify the biological functions in DEGs, Mercator4 analysis was performed to group the 7837 DEGs into all 31 biologically functional groups (Supplementary Data Table S6). These DEGs were mainly enriched in Enzyme classification (14.91%), RNA biosynthesis (9.55%), Solute transport (6.00%), Protein homeostasis (4.64%), and Cell wall organization (4.54%) (Fig. 4B, Supplementary Data Table S6), indicating that these pathways might play important roles in soybean shade tolerance. The DEG biological categories correspond to the GWAS-identified gene categories (I–V). Among them, GWAS category I corresponded to Solute transport, category II corresponded to Protein modification, category III corresponded to Enzyme classification, and category IV corresponded to Carbohydrate metabolism, there are still a large number of genes that have not been annotated, corresponded to category V (Fig. 4B). However, transcriptional analysis identified many more DEGs than GWAS genes (7837 DEGs versus 211 GWAS genes) in response to shade stress, especially involving more biological processes in transcriptional analysis, including those in Plant reproduction, Cellular respiration, and Phytohormone action, etc. (Fig. 4B, Supplementary Data Table S6).

To further identify the interaction relationship between DEGs, we constructed four DEG-PPI groups using the 7837 DEGs but not a one, because the limited number of genes in a single PPI analysis is 2000 DEGs, which explains why the total DEGs have to be run four times under random sampling (Fig. 4C). The results showed that 6635 (85%) DEGs were significantly interacting in the DEG-PPI groups (*P* = 1.0E−16^*^), forming a total of 74 990 interaction pairs (links = 1–398, Fig. 4C), indicating that DEGs interacted tightly for shade stress in the two parents. Five DEGs were considered as key hub DEGs (links = 163–398) forming a close association with other genes in each of the four DEG-PPI groups, in a total of 21 key hubs because the fourth group has two key ones with the same number of connections. From the 21 hubs, 17 further formed a general PPI group, and it was speculated that at least 85% DEGs can connect to form a huge overall PPI group as an overall DEG network (Fig. 4C). Thus, it can be expected that all the DEGs are in a DEG-PPI network. Surprisingly, 12 out of 17 key hub DEGs encoded ribosome subunits involving in gene expression (II), indicating that gene expression plays a core role in DEG-PPI networks.

From the above results, the DEGs covered a much wider range of functions than the GWAS-identified causal genes did. In comparisons among the 7837 DEGs, 12 STI and 17 RCL genes (two in common) were identified also in GASM-RTM-GWAS with five functional categories (1 I + 2 II + 4 III + 14 IV + 8 V) involved (Supplementary Data Tables S2, S3, and S7). In addition, GWAS genes and DEGs closely interacted; 26 out of 27 GWAS genes were identified also in the DEG-PPI networks, whereas 10 GWAS genes were not included in the GWAS-PPI network (Supplementary Data Table S7), indicating that there must be additional shade tolerance genes among the DEGs. These results indicated that 27 DEGs were representatives of the GWAS genes that regulate parental STI and RCL traits on day 30.

From the above, the 7837 DEGs, comprising wider functional categories of genes, with 6635 (85%) DEGs interacting together, compose a shade-tolerance-expressed gene network. In fact, 27 GWAS genes were included in the expressed gene network and more GWAS genes might be included if more transcriptome analyses were done. Therefore, the expressed gene network should be the total gene network of shade tolerance in soybean on day 30 in the present study.

### Chronological gene modules in shade tolerance development recognized from the GWAS-causal gene and differentially expressed gene network

The characteristics of GWAS-PPI and DEG-PPI networks are summarized as follows. (i) The shade tolerance gene network consists of many interacting genes involved in different functions related to shade tolerance. (ii) Most genes (nodes) have only one or several connections, and a few genes (hub genes) have a large number of connections with other genes, forming a scale-free network. (iii) The shade tolerance gene network is centered around genes involved in transcription and translation processes. (iv) The genes in the network not only have functional connections, but also have chronological order, i.e. they are expressed at a specific time in a fixed sequential process (e.g. 27 out of 211 genes were differentially expressed on day 30 after sowing/shading). (v) The gene network can be separated into chronological modules in its expression processes.

**Figure 5:**
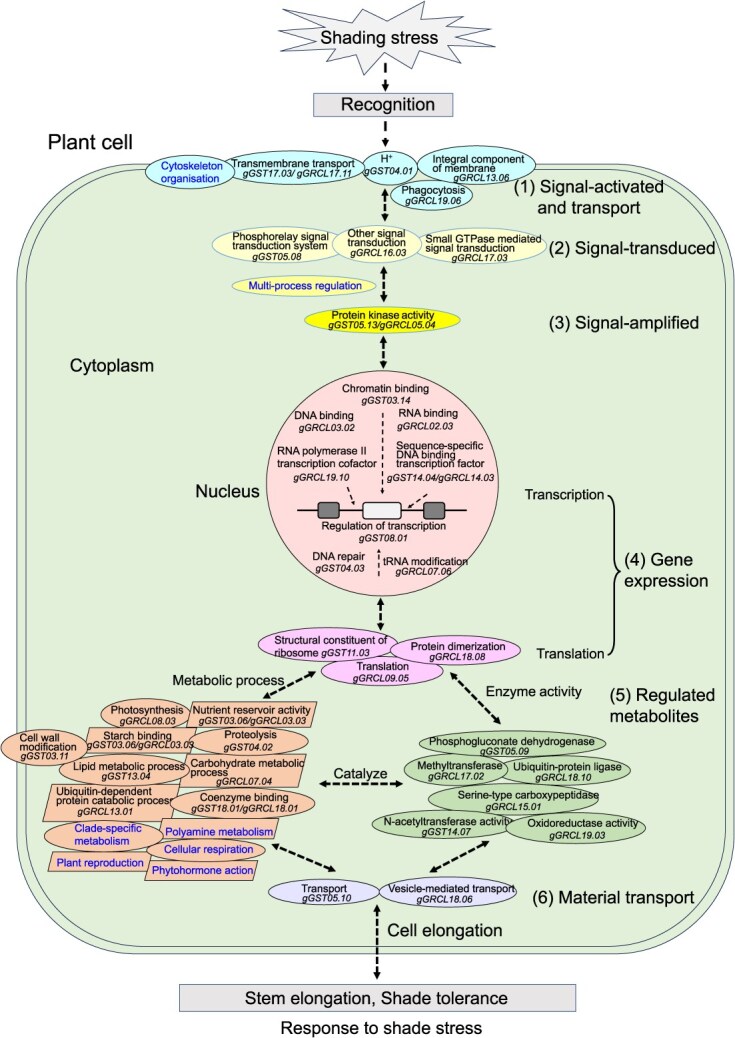
Possible module for genes in response to shade stress in the GZ-RIL population of soybean. Some organelles are not specified in this figure. The modules were described mainly with respect to GWAS-identified genes. A stage may involve multiple genes; here only one gene code is marked. Genes with unknown functions are not shown here; they may involve all the modules mentioned above and require further confirmation. (1)–(6) are the six chronological stage gene modules of the soybean response to shade stress. The biological processes marked in blue font are unique to DEGs.

The response of genes to abiotic stress is chronological rather than simultaneous in plants [26–28]. Thus, we speculated that shade stress elicits multilevel and sequential responses in soybean, involving numerous gene modules (Fig. 5, Supplementary Data Tables S2, S3, and S6). (i) Module 1, Signal activation and transport. At first, plants perceive shade stress, activate signal receptor genes (such as *gGRCL13.06* or *Glyma.13G328700*) and transmembrane transport protein genes (such as *gGST17.03/gGRCL17.1* or *Glyma.17G136400*) in the cell membrane to convert extracellular signals into intracellular signals. (ii) Module 2, Signal transduction. Then, the shade stress signal is further transformed and transmitted within the cell through intracellular signal transduction genes (such as *gGRCL17.03* or *Glyma.17G032100*). (iii) Module 3, Signal amplification. Some protein kinase genes (such as *gGST05.13/gGRCL05.04* or *Glyma.05G220900*) are phosphorylated to amplify the signal step by step. (iv) Module 4, Gene expression. The amplified signal enters the nucleus, induces gene transcription (such as *gGRCL19.10* or *Glyma.19G245100*) and translation (such as *gGST11.03* or *Glyma.11G027000*). (v) Module 5, Regulated metabolites. Subsequently, gene expression guides the encoding of various enzyme (such as *gGRCL15.01* or *Glyma.15G090300*) and protein genes (such as *gGST04.02* or *Glyma.04G028300*) required for tolerating shaded environments, as well as the genes that make up cellular chemical components (such as carbohydrates: *gGRCL07.04* or *Glyma.07G102900*; and lipids: *gGST13.04* or *Glyma.13G326100*), causing changes in intracellular substances. In addition, metabolic processes may accompany the catalytic effects of numerous enzyme genes (such as *gGST07.05* or *Glyma.07G186000*). (vi) Module 6, Material transport. After that, various products are secreted outside of the cell by means of vesicle-mediated transport (such as *gGRCL18.06* or *Glyma.18G202700*) and other modes of transport (such as *gGST05.10* or *Glyma.05G169500*). Finally, the plants perform shade tolerance/adaptability (realizing the rebalancing of the intracellular environment under stress conditions, enhancing the ability to tolerate shade stress, and achieving normal growth), or perform shade sensitivity (cell elongation, and then stem elongation). The module genes (mainly GWAS genes) were activated one by one and level by level in the Modules 1-6. In addition to GWAS genes, DEGs also participate in Modules 1–6, including seven biological processes in the Mercator4 classification that are unique DEGs (Fig. 5, Supplementary Data Table S6). In the developmental processes, the responses of DEGs to external stimuli are involved in Modules 1–3; RNA processing and protein biosynthesis are involved in Module 4; enzyme classification, photosynthesis, carbohydrate metabolism, and lipid metabolism participate in Module 5; and vesicle trafficking and solute transport participate in Module 6. Modules 1–6 may work back and forth and even cross over among the regulatory phenomena to maintain metabolic balance through dynamic positive and negative feedback corrections. The correlations and causal relationships of genes enable biochemical information to be transmitted between related genes or systems.

Obviously, the gene composition of the chronological modules needs further exploration, although some 12 major key genes are listed here according to the following criteria: (i) GWAS-detected STI and/or RCL main-effect ones, especially those with a large contribution (*R*^2^ ≥ 1.5%) and shared by both indicators; (ii) identified by both GASM-RTM-GWAS and transcriptome analysis as the preference; and (iii) linked in both GWAS- and DEG-PPIs, especially the major hub genes as the preference (Table 2, Supplementary Data Tables S2, S3, and S7). The 12 key genes that were nominated are the following (Table 2): *gGST17.03/gGRCL17.11* (*Glyma.17G136400*) in Module 1, *gGST05.13/gGRCL05.04* (*Glyma.17G032100*) in Module 2, *gGST05.13/gGRCL05.04* (*Glyma.05G220900*) in Module 3, *gGST14.04/gGRCL14.03* (*Glyma.14G070000*) and *gGST11.03* (*Glyma.11G003700*) in Module 4, *gGST11.02/gGRCL11.01* (*Glyma.11G002600*), *gGST03.06/gGRCL03.03* (*Glyma.03G063000*) and *gGST18.01/gGRCL18.01* (*Glyma.18G057900*) in Module 5, *gGRCL18.06* (*Glyma.18G202700*) in Module 6, and *gGST05.06* (*Glyma.05G10200*), *gGRCL05.01* (*Glyma.05G206400*), *gGST19.04* (*Glyma.19G225200*) with unknown function but with large contribution in GASM-RTM-GWAS. Briefly, soybean shade tolerance is a response process centered about gene activation and expression, in which genes involved in various biological processes interact together. The suggested key genes are only a first part and are recommended as a starting point for further exploring the complete picture of the shade tolerance gene network.

**Table 2:** Key STI and RCL genes in the GZ-RIL population.

Stage of module	Code	Gene ID	*R* ^2^ (%)^a^	Functional annotation of the gene	STI main effect	RCL main effect	LC^b^	PPI hub gene^c^	DEG^d^	
							
(1) Signal-activated	*gGST17.03/ gGRCL17.11*	*Glyma.17G136400*	0.61/	Transmembrane transport	√	√	√			
			2.54							
(2) Signal-transmitted	*gGRCL17.03*	*Glyma.17G032100*	0.38	Small GTPase mediated signal transduction		√				
(3) Signal-amplified	*gGST05.13/ gGRCL05.04*	*Glyma.05G220900*	2.63/	Protein kinase activity	√	√	√			
			0.17							
(4) Gene expression (translation)	*gGST14.04/ gGRCL14.03*	*Glyma.14G070000*	1.76/	Sequence-specific DNA binding transcription factor activity	√	√	√			
			2.54							
(4) Gene expression (transcription)	*gGST11.03*	*Glyma.11G003700*	2.38	Structural constituent of ribosome	√		√	20	√	
(5) Regulated metabolites	*gGST11.02/ gGRCL11.01*	*Glyma.11G002600*	0.41/	Protein binding	√	√	√	12		
			4.89							
	*gGST03.06/ gGRCL03.03*	*Glyma.03G063000*	0.47/	Starch binding		√	√		√	
			3.23							
	*gGST18.01/ gGRCL18.01*	*Glyma.18G057900*	0.14/	Coenzyme binding	√	√			√	
			0.05							
(6) Transport	*gGRCL18.06*	*Glyma.18G202700*	0.14	Vesicle-mediated transport		√				
Unknown	*gGST05.06*	*Glyma.05G102000*	1.74	Unknown	√		√	15		
	*gGRCL05.01*	*Glyma.05G206400*	2.53	Unknown		√	√	12		
	*gGST19.04*	*Glyma.19G225200*	1.53	Unknown	√		√		√	

a *R*  ^2^ is the contribution rate of a gene.

b Large-contribution (*R*^2^ ≥ 1.5%) genes in STI or/and RCL.

c A gene that interacted frequently with other genes (links ˃10) is considered a hub gene. Listed is the number of genes that interacted with a specific hub gene in the PPI network.

d Differentially expressed genes in G_S30_ versus G_CK30_ or/and Z_S30_ versus Z_CK30_.

## Discussion

### Shade tolerance as a complex trait conferred by interrelated causal and expressed gene networks

In the forward genetic study, the STI and RCL gene systems were detected from the GASM-RTM-GWAS, which composed an interrelated regulatory gene network with a total of 211 GWAS genes, whereas in the reverse genetic study transcriptome analysis identified 7837 DEGs, many more than those from GWAS. The latter contained part of the former’s genes (27) and might cover all the GWAS genes if many more transcriptome analyses had taken place, according to reference [11]. The large difference in identified genes between the forward and reverse genetic analyses is due to the following factors: (i) the expression of a GWAS gene involves a series of steps and their genes; (ii) the GWAS-identified genes only involve two traits (STI and RCL), but more traits may be involved in shade responses in transcriptome analysis; and (iii) transcriptome analysis is a chronological/spatial process but done only once, on the 30th day – more such analyses should be done. Thus, the gene network identified by reverse genetics may cover genes from both the forward and the reverse genetics-identified gene network.

Between the forward and reverse parts of the gene network, the GWAS-identified genes were fewer, mainly hub ones in the joint gene network, while the reverse genetics-identified genes were much more numerous, mainly linked to the hubs. From the forward and reverse gene network, the sequential shade tolerance development occurs in a total of seven stages from signal activation through material output to plant response (six module stages plus the final plant response stage), and their gene modules and functions were assembled. Therefore, shade tolerance is a complex trait covering multiple developmental stages, and is conferred by interrelated causal and expressed genes.

### Strategies and procedures in exploring the shade tolerance gene network

The shade tolerance in soybean has been reported to be a complex quantitative trait [10, 11, 13]. The present study started from identifying the gene systems of the external STI and internal RCL indicators, then used PPI to explore the functional categories and interrelationships among the genes. On the other hand, the reverse genetic analysis was conducted to identify the expressed gene system (DEGs), then PPI analysis was also done. The results indicated that between the GWAS-identified genes and transcriptome-identified genes, the latter may cover the former if a time series of transcriptome analyses can be completed fully. Accordingly, the gene modules and functions of chronological shade tolerance development stages were assembled from signal activation through signal transmission to plant response. Finally, the 12 key shade tolerance genes were identified as starting points for further gene function validation study. Previously, most of the studies on quantitative traits have been on individual gene(s) [29–32]. However, the present objective concerned the gene system/network, trying to identify the complete shade tolerance system, then to pick up the key ones for further research. Although some conclusions of this study are inferential, they are reasonable and simply verified by GWAS and transcriptome analysis. Therefore, the results of this study may provide an valuable basis for the further function verification of shade tolerance genes and molecular network research.

However, in the analytical processes some points are to be emphasized. (i) Identifying the forward gene system is the base for further analysis. Especially, the GASM-RTM-GWAS procedure can directly and thoroughly detect genes and their alleles related to STI and RCL. The statistical reasons and merits for the powerfulness, efficiency, and effectiveness of the GASM-RTM-GWAS procedure have been explained in previous studies [13]. (ii) Transcriptome analysis explored a great number of DEGs. If the GWAS genes are activated to cause differential expression, its expression level will be ten or hundred times greater than before activation. As indicated by many reports [11, 33–37], the transcriptome has time–space limitations. In the present study, only one date specimen (day 30) was analyzed for the transcriptome, so the GWAS-identified genes were not completely identified in this single analysis. It can be expected that all the GWAS genes should be activated for all the possible correspondingly expressed DEGs. This point is to be explored further in later studies. (iii) The GWAS gene and DEG functional annotation is very helpful in understanding the involved biological processes and functions for each GWAS gene, and from this we understand the genetic system of shade tolerance is a complex gene network. And we understand why the STI and RCL indicators are significantly correlated (*r* = 0.54^**^) but only 7 are shared out of the 211 because the gene function annotations of the two indicators were similar. (iv) The GWAS-PPI is the interacting gene network between the two traits, whereas the DEG-PPI is the interacting DEG network for all genes related to shade stress. That is, if the transcriptome analysis is performed enough times, the DEG-PPI network will include the GWAS-PPI network. However, the current PPI procedure is limited in sample size (2000) and needs to be extended to a larger sample, even more than 10 000–20 000. (v) The DEG-PPI network suggests that different shade tolerance developmental stages have their own unique interaction genes. Thus, separation of the GWAS genes and DEGs into chronological modules is relevant for understanding the processes and mechanisms of shade tolerance. Unfortunately, we nominated the key genes for various chronological stages, but could not describe all the genes/DEGs in the shade tolerance gene network due to lack of all the detailed information on the genes/DEGs. Meanwhile, systems biology suggests that signal transduction and various metabolic responses are not unidirectional, but a complex process that undergoes continuously positive and negative feedback for correction.

From the above, GWAS and the transcriptome could provide entrance information for understanding the genetic mechanism of shade tolerance, while the chronological modules are to be further explored and connected for a thorough understanding and adjustment of shade tolerance in soybean.

## Materials and methods

### Plant materials and field experiments

An RIL population with 246 F_2:5:7_ lines was developed through single-seed descendant from the cross of Gongdou-7 (shade-tolerant parent) × Zhizidou (shade-sensitive parent), designated GZ-RIL [10, 13]. The RILs with their parents were evaluated in a shading net room (42 m × 16 m × 3.5 m) with 30% light reduction and paralleled in a field without shading at the experimental station of Guangxi Academy of Agricultural Sciences (23.17°N, 108.27°E) in 2017–19. Both sets were arranged in the same randomized complete block design with three replications, one row plot with 13 plants in a row of length 1.2 m with 0.45 m between rows. Each shading and non-shading replication was paralleled to minimize the soil difference. In each plot, five middle plants were measured for plant height (PH) and main stem node number (NN) on day 50 after sowing. The STI was calculated as: STI = (PH_shade_/PH_CK_ + AINL_shade_/AINL_CK_)/2, where subscript CK indicates the control condition and AINL is average internode length (=PH/NN) [10, 13].

### Relative pith cell length

The middle segment of the penultimate internode on the main stem was sampled for the central three plants per plot on day 50 after sowing in 2018. The specimens were fixed and paraffin-sliced as described in [11] and then photographed using light microscopy (Ni-E, Nikon, Yokohama, Japan). The cell length data of the RILs and parents were collected.

To observe pith cell length, a longitudinal section specimen was randomly selected, and 8–10 different micrographs were captured, with all pith cell lengths measured in each micrograph using the software NIS-Elements D 4.11.00. RCL values were calculated with Excel 2019 software for each plot of the RILs and parents.


\begin{align*} \textrm{RCL}=\textrm{CL}_{\textrm{shading}}/\textrm{ACL}_{\textrm{CK}} \end{align*}

where CL_shading_ is the average length of all pith cells in the same micrograph under 30% shading, and ACL_CK_ is the average length of all pith cells (multiple photos) of the control. Due to the labor-intensive anatomical analysis, five randomly selected RCL values were used for each RIL in only one replicate for further statistical and association analyses.

### Statistical analysis of phenotypic data

The STI and RCL data were calculated using Excel 2019 software. Analysis of variance was performed using SAS 9.4 software (SAS Institute Inc., Cary, NC). The broad-sense heritability was estimated according to the following equation: *h*^2^ = *σ*^2^_g_/ (*σ*^2^_g_ + *σ*^2^_ge_/*n* + *σ*^2^_ε_/*nr*) × 100% for STI and *h*^2^ = *σ*^2^_g_/(*σ*^2^_g_ + *σ*^2^_ε_/*r*) for RCL, where *σ*^2^_g_, *σ^2^*_ge_, and *σ*^2^_ε_ represent genotype, genotype × environment, and random error variance, respectively, *n* is the number of environments, and *r* is the number of replications [38]. The genotypic coefficient of variation (GCV) was calculated as GCV = *σ*_g_/*μ*, where *μ* is the estimated population mean value [39].

### Genotyping of the GZ-RIL population

Genomic DNA was extracted from fresh and tender leaves of the 246 RILs and their parents using the CTAB method. Whole-genome resequencing was carried out using Illumina HiSeq2000 (Annaroad Gene Technology (Beijing) Co., Ltd, China). The sequencing depth was ~2.0× for the RILs and ~5.0× for the parents. All sequence reads were aligned against the reference genome *Wm82.a2* in SoyBase (http://www.soybase.org) using BWA v0.7.9a with default parameters. The identification method for high-quality SNPs is described in reference [13].

All SNPs in a gene were assembled directly into a GASM using the RTM-GWAS program [13, 14]. A GASM represents a gene with SNPs and each GASM contains two alleles.

### Identification of shade tolerance index and relative pith length gene systems using GASM-RTM-GWAS

At the first stage of GASM-RTM-GWAS, the linear regression under a single-locus model at *P* ≤ 0.05 was used to preselect markers for STI and RCL from all GASMs. At the second stage, the preselected GASMs were applied to identify associated genes and allele effects using stepwise regression under a multilocus model with and without RILs × environment interaction for STI and RCL at *P* ≤ 0.05, with total contribution less than *h*^2^, respectively. The multilocus model (built-in experiment-wise error control) is equivalent to the Bonferroni criterion under the single-locus model (*P*/*m*, where *P* is significance at 0.05 and *m* is the number of markers involved) [14]. A gene with contribution (*R*^2^) ≥1.5% was considered a large-contribution (LC) gene, while a gene with *R*^2^ <1.5% was considered a small-contribution (SC) gene. However, the boundary is not fixed, but varies according to the inflection point of the gene contribution curve [14].

The data on the estimated allele effects for all the identified markers were organized as an STI or RCL gene–allele matrix to calculate their recombination potential (see He *et al*. [14] for detailed methods).

### Gene function annotation and protein–protein interaction analysis

The identified STI and RCL genes were annotated with their functions according to the GO annotations in SoyBase (http://www.soybase.org).

Protein sequences of STI and RCL genes were copied from the Phytozome database (http://phytozome.jgi.doe.gov). These protein sequences were submitted to the *Glycine max* section of STRING v12.0 (http://string-db.org) [40] to generate the PPI relationship. The minimum required interaction score (confidences in 0–1) was set to ≥0.18 as an approximate probability of a predictive link between two proteins.

### Transcriptome analysis and differentially expressed gene identification

The main-stem tips of three plants per plot of the two parents Gongdou-7 and Zhizidou under shading (S) versus open field (CK) conditions were collected from the three replications, and then immediately frozen in liquid nitrogen on day 30 after sowing. The specimens were sent to Gene Denovo Biotechnology Co. (Guangzhou, China) for RNA extraction and sequencing using the Illumina HiSeq2500 program. The sequencing data were designated as G_S30_ and Z_S30_ under shading stress and G_CK30_ and Z_CK30_ under the open field condition for Gongdou-7 and Zhizidou on day 30, respectively. After the removal of low-quality reads, the clean reads were aligned to the Wm82.a2 reference genome [41–43]. The fragments per kilobase million (FPKM) values were calculated to estimate gene expression levels [44–47]. DEGs were identified in each pair of samples using the criteria of *P* ≤ 0.05 and |log_2_fold change| ≥0.807 [48].

In addition, for verification of the transcriptome results, quantitative real-time PCR (qRT–PCR) was conducted in parallel to detect the expression levels of three randomly selected GWAS-identified genes for each parent. The identification method of qRT–PCR was as described [11], and all primers are listed in Supplementary Data Table S8.

### Functional annotation and protein–protein interaction of identified differentially expressed genes

The DEGs were functionally analyzed using Mercator4 v.6 (http://www.plabipd.de/portal/mercator-sequence-annotation) [49]. The Mercator4 functional annotations were designed as a hierarchical framework with each child node term being more specialized than its parent node term, including 31 top-level categories.

The maximum protein capacity predicted by PPI the network is 2000 in the STRING database. To explore the interactions among DEGs, we randomly and evenly divided the 7837 DEGs into four groups for PPI network prediction. Accordingly, an overall PPI network was established using the top five DEGs from the four groups that interact with other DEGs. The processes can of constructing the PPI networks of DEGs be referred to the PPI networks construction method of GWAS genes mentioned above (http://string-db.org).

## Supplementary Material

Web_Material_uhae333

## Data Availability

All relevant data in this study are provided in the article and its supplementary figure and table files.
