# Supplementary material for: Characterization of shade tolerance gene network in soybean revealed by forward integrated reverse genetic studies
Source: Hortic Res. 2024 Nov 26;12(3):uhae333. doi: 10.1093/hr/uhae333 (PMC11879493; doi:10.1093/hr/uhae333)
Supplement: Web_Material_uhae333 [file web_material_uhae333.zip › Supplementary Figure.docx]

**Supplemental information**

**Characterization of shade-tolerance gene network in soybean revealed by forward integrated reverse genetic studies**

Yanzhu Su^1^, Yongpeng Pan^1^, Weiying Zeng^2^, Zhenguang Lai^2^, Pengfei Guo^1^, Xiaoshuai Hao^1^, Shengyu Gu^1^, Zhipeng Zhang^a^, Lei Sun^1^, Ning Li^1^, Jianbo He^1^, Wubin Wang^1^, Guangnan Xing^1^, Jiaoping Zhang^1^, Zudong Sun ^2, *^ and Junyi Gai ^1, *^


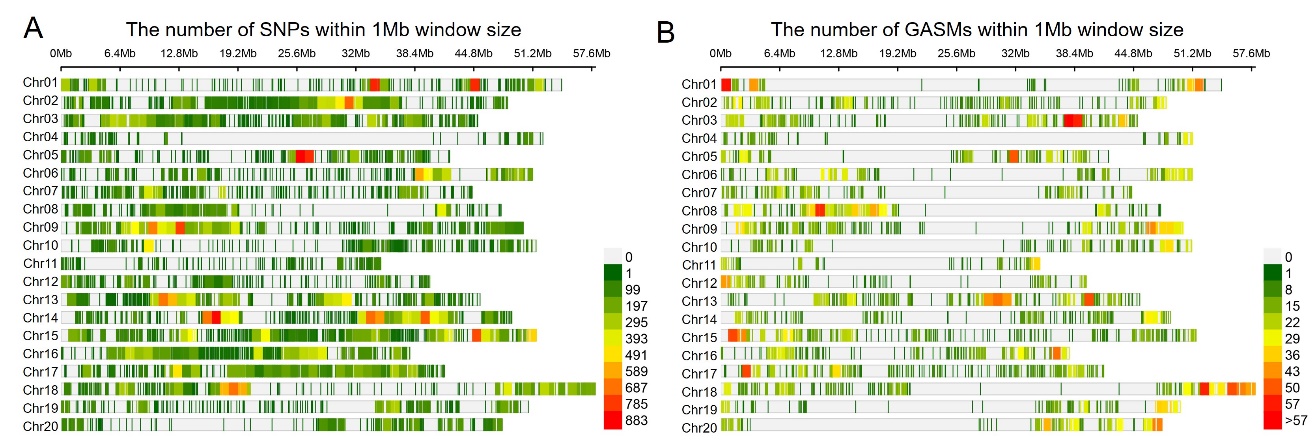


**Supplementary Figure 1** Distribution of single nucleotide polymorphism (SNP) markers and gene-allele sequence markers (GASMs) in the GZ-RIL population

(A) Genome-wide distribution of SNPs in GZ-RIL population.

(B) Genome-wide distribution of GASMs in GZ-RIL population.


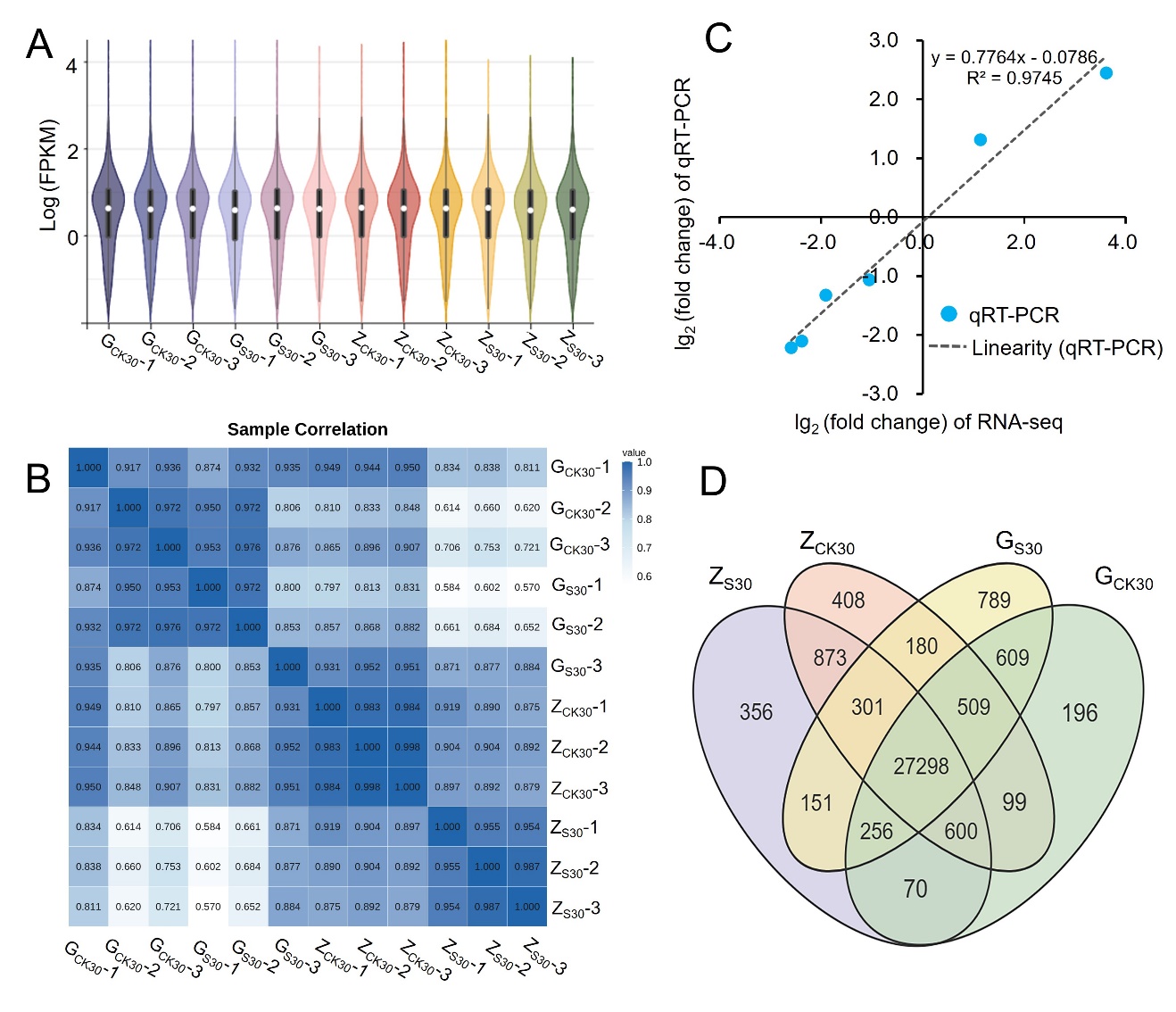


**Supplementary Figure 2** Transcriptome analysis of the two parents

(A) Violin plot of the sample genes’ expression.

(B) Correlation diagram of gene expression.

(C) Correlation between qRT-PCR and RNA-seq data.

(D) Venn diagram of all expressed genes in two parents on day 30.
